# Knowledge, attitude, and practice (KAP) of antimicrobial prescription and its resistance among health care providers in the COVID-19 era: A cross sectional study

**DOI:** 10.1371/journal.pone.0289711

**Published:** 2023-08-10

**Authors:** Nader Nemr, Rania M. Kishk, Noha M. Abu Bakr Elsaid, Nageh Louis, Eman Fahmy, Sally Khattab

**Affiliations:** 1 Endemic and Infectious Diseases Department, Faculty of Medicine, Suez Canal University, Ismailia, Egypt; 2 Microbiology and Immunology Department, Faculty of Medicine, Suez Canal University, Ismailia, Egypt; 3 Public Health, Community, Environmental and Occupational Medicine, Faculty of Medicine, Suez Canal University, Ismailia, Egypt; 4 Department of Basic Medical Sciences, Faculty of Medicine, King Salman International University, South Sinai, Egypt; 5 Internal Medicine Department, Faculty of Medicine, Suez Canal University, Ismailia, Egypt; Lahore Medical and Dental College, PAKISTAN

## Abstract

Antimicrobial resistance (AMR) is considered as a global health and development threat. During COVID-19 pandemic, there has been an increase in antimicrobial resistance. Health care providers (HCPs) play the main role in facing antibiotic resistance because they have the authority to prescribe antibiotics during clinical practice as well as in promoting patients’ compliance with therapies and avoid self-medication. So, this study will serve as an important source of information in context with Covid19 pandemic in Egypt. The data was collected using a validated standardized self-administered online questionnaire compromised of four sections: socio-demographic data of the HCPs, the general knowledge on antibiotics and AMR, the HCP attitude towards antibiotic prescription and AMR and the practice in applying the appropriate antibiotic prescription. Most of HCPs (93.7%) recorded good knowledge level about antibiotic prescription and antimicrobial resistance with mean score of knowledge13.21 ± 1.83. About 79% of HCPs recorded a positive attitude towards proper antibiotic prescription with mean score of attitudes 63.02 ± 7.68. Fifty four percent of HCPs demonstrated a good level of practice with mean score of practice 9.75 ± 2.17. In conclusion, HCPs in our study have a good level of knowledge and attitude about antibiotics. However lower level of proper practice towards the problem of AMR in COVID19 era was noticed. Implementation of effective policies and guidelines is crucial to evaluate the antimicrobial use especially in the COVID-19 era to reduce the unintended consequences of the misuse of antibiotics and its impact on AMR.

## Introduction

Antimicrobial resistance (AMR) is considered as a global health and development threat. Bacterial infections that are unsuccessfully treated due to antibiotic resistance was reported to cause mortality rate of at least 700,000 people per year worldwide and are expected to kill 10 million people per year by 2050, at a cost of US$100 trillion in lost output to the global economy. The World Health Organization (WHO) has announced AMR as a major global problem and started a global action plan to combat AMR [1]. One of the parts of this action plan is to enhance knowledge and surveillance skills on AMR by doing global antimicrobial resistance surveillance system. The WHO’s global strategy emphasized the need for proper antibiotic usage as well as the critical role of healthcare providers [2].

Corona virus disease (COVID-19), which is caused by the severe acute respiratory syndrome coronavirus 2 (SARS Cov2), had caused 764,474,387 confirmed cases, including 6,915,286 deaths as reported on 26 April 2023 by WHO [3]. During COVID-19 pandemic, there has been an increase in antimicrobial resistance globally [4, 5]. The COVID-19 pandemic resulted in a decrease of patient screening exams and admissions, healthcare personnel and a surge in misinformation. In addition to the financial crisis that led to disruption of healthcare system [6–8]. The disruption of surveillance and vaccinations may cause an increase of infections and leads to the overuse of antimicrobials [9].

A large proportion of COVID-19 patients need hospitalization and are at a great risk of developing hospital-acquired bacterial and fungal infections [10, 11]. Many recommendations encourage empirical use of antibiotics in severely ill patients. Secondary infections in COVID-19 patients are known to be associated with negative health outcomes [12]. The WHO estimates that in 2020, Egypt’s daily consumption of antibiotics per 1000 people was 38.5 DDD. This was the WHO Eastern Mediterranean Region’s second-highest consumption level after Iran [13]. In comparison to 2019, the use of outpatient antibiotics rose by 53.80% in 2020. In 2020, 2021, and the first half of 2022, respectively, 91.04%, 83.05%, and 73.52% of COVID-19 outpatients received at least one antibiotic prescription. More than 55% of COVID-19 outpatients received a prescription for at least one antibiotic. The three antibiotics that were most frequently prescribed were doxycycline, amoxicillin, and azithromycin [14].

Initial studies had raised concerns about the long-term impact of the COVID-19 pandemic on prevalence and predictors of antibiotic prescription in COVID-19 Patients. These studies had reported that antibiotics are commonly prescribed to patients with COVID-19 despite the relatively low prevalence of bacterial co-infection (less than 10%). A previous study in India found that bacterial and fungal super infections were uncommon in hospitalized COVID-19 patients. Most of super infections were nosocomial and caused by Extensively Drug Resistant (XDR) bacteria because of antibiotic overuse at that time. Poor Infection control and empirical over-use of broad-spectrum antimicrobials are two practices that should be avoided to prevent future epidemics of XDR organisms [11].

Another study conducted in France found that general practitioners (GPs) tended to overprescribe broad-spectrum antibiotics for extended periods of time for COVID-19 patients as well as other viral infections. They had a higher, but not statistically significant, ratio of azithromycin over all other antibiotics [15].

Inappropriate antibiotic prescriptions in COVID-19 patients can cause major complications such as increased bacterial resistance, adverse drug reactions, clostridioides difficile infections and renal impairment [16, 17]. As a result, several organizations have implemented antibiotic stewardship programs [18]. A retrospective cohort study that was conducted in Spain in 150 hospitals included patients who were discharged after hospitalization or died due to COVID-19, reported that 4769 (34.2%) of patients were inappropriately prescribed antibiotics [19].

Health care providers (HCPs) play the main role in facing antibiotic resistance because they have the authority to prescribe antibiotics during clinical practice as well as in promoting patients’ compliance to therapies and avoid self-medication [20]. Unfortunately, the inappropriate and overuse of antibiotic prescriptions by health care providers has been found to be a problem [21]. Some studies have documented that the real indication for antibiotic treatment; the choice of antibiotic class or the dosage may be incorrect in 30–50% of patients even in hospitals. In the intensive care units, inappropriate prescribed antibiotics were found to be in 30–60% of cases. Studies in China and Europe has revealed that 60%, 40% of antibiotics prescriptions were without any indication respectively [22, 23].

Minimizing the use of antibiotics is the best way to stop the spread of AMR, and this can be done by changing prescribing practices. Evidence-focused questionnaires like knowledge, attitude, and practices (KAP) surveys can be used to analyze the factors influencing medical doctors’ prescribing behaviors [24].

In a systemic review published in 2017, discussing the antimicrobial resistance in Africa, reported that more than a 33% of the African countries did not have recent published data about AMR although they noticed high level of antibiotics resistance in these African countries [25]. This review recommends the necessity to improve the standardization and quality of the microbiological identification and susceptibility testing methods to facilitate the national and international organizations to monitor the extent of the AMR problem.

In Egypt, antimicrobial therapy was mostly prescribed empirically. As reported in the Egyptian National Action Plan for Antimicrobial Resistance, only in 4% of antimicrobials prescribed based on culture for community acquired infections and in 18% of antimicrobials prescribed for Hospital Acquired Infections. Inappropriate prescriptions may be caused by knowledge gaps in antibiotics uses and resistance development as well as a lack of legal characteristics in antibiotic distribution [26].

There were a limited number of previous KAP studies, and most were conducted before the pandemic. Prescribers may change their practice only when their knowledge, attitudes, thoughts, skills, and knowledge are integrated with each other, resulting in a reduction in antibiotic prescribing. This will in turn reduce the development and spread of infections that are resistant to antibiotics [27].

Recently, an Egyptian study published in 202 reported a good level of knowledge about antibiotics with low levels of positive attitude and proper practice towards the problem of AMR before COVID-19 pandemic. They also reported that only (15.4%) of the Egyptian HCPs referred to national antibiotic guidelines as the main reference of data about antibiotic resistance and stewardship program contrasted with (19.8%) who used the international guidelines which are considered unacceptable low rate of using both national and international guidelines [28].

It is a hot issue to resolve the problem of antibiotic usage during COVID-19 pandemic among prescribers to limit the development of resistance. To the best of our knowledge, this is the first national study that identifies knowledge, characterizes practices, and describes the attitude of Egyptian prescribers towards antibiotic use during COVID-19 pandemic. So, this study will serve as an important source of information in context with Covid19 pandemic for other health care professionals to improve practice of antibiotic prescription. Moreover, this study will help health officials and policy makers in designing an evidence-based education and advocacy plan on prevention of antibiotic resistance in Egypt.

## Patients and methods

### Study design

A cross sectional analytic online based study was conducted to assess KAP of health care providers (HCPs) toward antibiotics prescription and its resistance in context with COVID19 pandemic in Egypt. We hypothesized that HCPs had good knowledge with less positive attitude and malpractices regarding antibiotics prescription during COVID-19 pandemic.

### Study setting

The study was conducted in governmental and private hospital in Egypt, officially the Arab Republic of Egypt. The Arab Republic of Egypt spans the northeast corner of Africa. Egypt is one of the most populous countries in Africa and the Middle East. The estimated population is about 99.38 million with a population density of Egypt as a whole is 48 people per km^2^ as reported in 2018 [29]. Egypt, a country with a lower-middle income, is dealing with an urgent public health crisis that is a lack of retention of its health workforce. Egypt includes 27 governorates.

### Study population

Healthcare Providers including physicians and dentist working governmental and private hospitals in Egypt dealing with patients and handling antibiotics for the treatment of cases. Participants were recruited to study during the period between January 2022 and July 2022. According to the Egyptian Medical Syndicate, the Ministry of Health (MoHP) had 213,000 licenced physicians as of September 2020. However, of those registered, only two fifths, or 82,000, actually practice in the country [30].

#### Inclusion criteria

Physicians aged from 25 to 65 years, including medical doctors (MD), internist and various clinical specialties (surgery, ENT, urology, gynecologist, anesthesiologist, orthopedist, family medicine, tropical medicine and emergency medicine) were included from the study.

#### Exclusion criteria

Radiologists, and basic sciences doctors were excluded. Egyptian physicians working outside Egypt or in annual leaves.

### Sample size and technique

Convenience sampling method was implemented. The sample size was Calculated to be 317 participants based on a reported previous proportion of adequate knowledge among Egyptian health care physicians (71.6%) with a margin of error (5%) [28]. Then after adding 10% non-response rate, the sample was estimated to be 350.

## Methods

### Data collection tool

The data was collected using a validated standardized self-administered online questionnaire used before in Egypt [28]. The questionnaire was available in English language and comprises of four sections. The first section consisted of socio-demographic data of the health care providers. The second section assessed the general knowledge on antibiotics and its resistance. It consisted of eighteen questions. Each question was answered by either yes, no, or do not know. The third Section consisted of seventeen questions to determine the HCP attitude towards antibiotic prescription and AMR. Responses for the attitude section were recorded on the 5-point Likert scale as follows: strongly agree, agree, neutral, disagree, and strongly disagree. The fourth section included four questions to identify the participants’ practice in applying the appropriate antibiotic prescription. The structured questionnaire was merged into online Google form. The URL link was distributed to HCPs through all social media platforms including them such as WhatsApp, Facebook, and Messenger from the start of January 2022 till the completion of required sample. The title, aim and objectives of the study were clarified in the begining of the questionnaire form. A clear statement was included in the beginning of the questionnaire form that completion of it indicates participant’s consent to participate in the study. The data of the participants was anonymous to the authors. We have activated the option limit to one response to avoid more than one response from the same participant.

### Data management

The data was downloaded from the Google form and checked for any missing answers. SPSS version 23 was used to analyze data. Mean and S.D were calculated for quantitative variables. Categorical data was analyzed by computing percentages. Categorical data were compared using Chi-square test (χ2test). P-value of ≤0.05 was considered statistically significant. The associations between the binary outcome variables (good versus poor knowledge and favorable versus unfavorable attitude) of the respondents on antibiotics usage and resistance with the independent socio-demographic characteristics of participant such as age, gender, level of education and work institutions of the respondents were analyzed.

### Regarding the scoring system of the questionnaire

For knowledge score, a score of “1” was assigned to every correct answer and ‘0’ for every wrong answer. The total score was added, and it ranged from 0 to18. A cut off value for good knowledge level is equal to or above 60% of total marks while, those who scored below that was defined as “poor” knowledge on antibiotics use and antibiotic resistance. Similarly, for the attitude related questions, a score of 5 to 1 was assigned to each question according to the selected answer on the Likert scale then the total score was added. The total score ranged from 17 to 85. Those who scored equal or above 60% of marks were considered as “favorable attitude” but when the scores were less than that, it was considered as “unfavorable attitude towards antibiotics prescription and AMR. For assessing practice, antibiotic prescription practice was calculated by giving one point for each selected step. The total score ranged from 1 to 4. Practice level ≥60% of marks was categorized as an appropriate practice.

### Ethical approval

The study was approved by the Research Ethics Committee, Faculty of Medicine, Suez Canal University (Research 4605#).

### Consent to participate

The structured questionnaire was merged into online Google form. The URL link was distributed among healthcare providers. A clear statement was included in the beginning of the questionnaire form that completion of it indicates participant’s consent to participate in the study.

### Consent to publish

A clear statement was included in the beginning of the questionnaire form that completion of it indicates participant’s consent to publish the results of this study.

## Results

The current study included 350 Egyptian health care providers (HCPs). Below half of HCPs (42.8%) live in Ismailia city while the remaining 57.1% were from another eleven governorates which are (Suez (18.5%), Cairo(14.2%), Sharkya (7.1%), North Sinai(4.5%), Portsaid(2.8%) Menofia(2.5%),Qena(1.5%),Minya(1.5%), Gharbia(1.5%), Dakahlya (1.5%), and Alexandria(1.5%). Males represented half of the sample (50.3%). The mean age of participants was 34.75 ± 7.41. Almost all the participants were physicians and only one participant was dentist. Near a quarter of the studied participants (24.6%) had obtained Medical Decorate (MD) degree while 34% had master’s degree. The highest percentage of HCPs (171 HCPs) were working in university Hospitals (48.9%), while the lowest percentage (12 HCPs) were worked in private clinics (3.4%). Three quarters of HCPs (248) (70.9%) didn’t attend any training/ educational course for antibiotics prescription (Table 1).

**Table 1:** Distribution of the studied cases according to demographic data (n = 350).

	No.	%
**Sex**		
Male	176	50.3
Female	174	49.7
**Age**		
<30	88	25.1
30-<40	189	54.0
40-<50	57	16.3
≥50	16	4.6
Mean ± SD.	34.75 ± 7.41	
Median (Min.–Max.)	34.0 (21.0–65.0)	
**Work Institution**		
Primary Health Care	17	4.9
Ministry of Health and Population Hospital	65	18.6
Health Insurance Organization	59	16.9
University Hospital	171	48.9
Private Hospital	26	7.4
Private clinic	12	3.4
**Educational Level**		
Bachelor	70	20.0
Diploma	20	5.7
Master	119	34.0
Doctorate	86	24.6
Fellowship	55	15.7
**Work Experience**		
Less than 1 year	51	14.6
1–5 years	55	15.7
5 years—10 years	84	24.0
More than 10 years	160	45.7
**Did you attend any training/ educational course for antibiotics prescription**		
Yes	102	29.1
No	248	70.9

SD: **Standard deviation**

**Fig 1:**
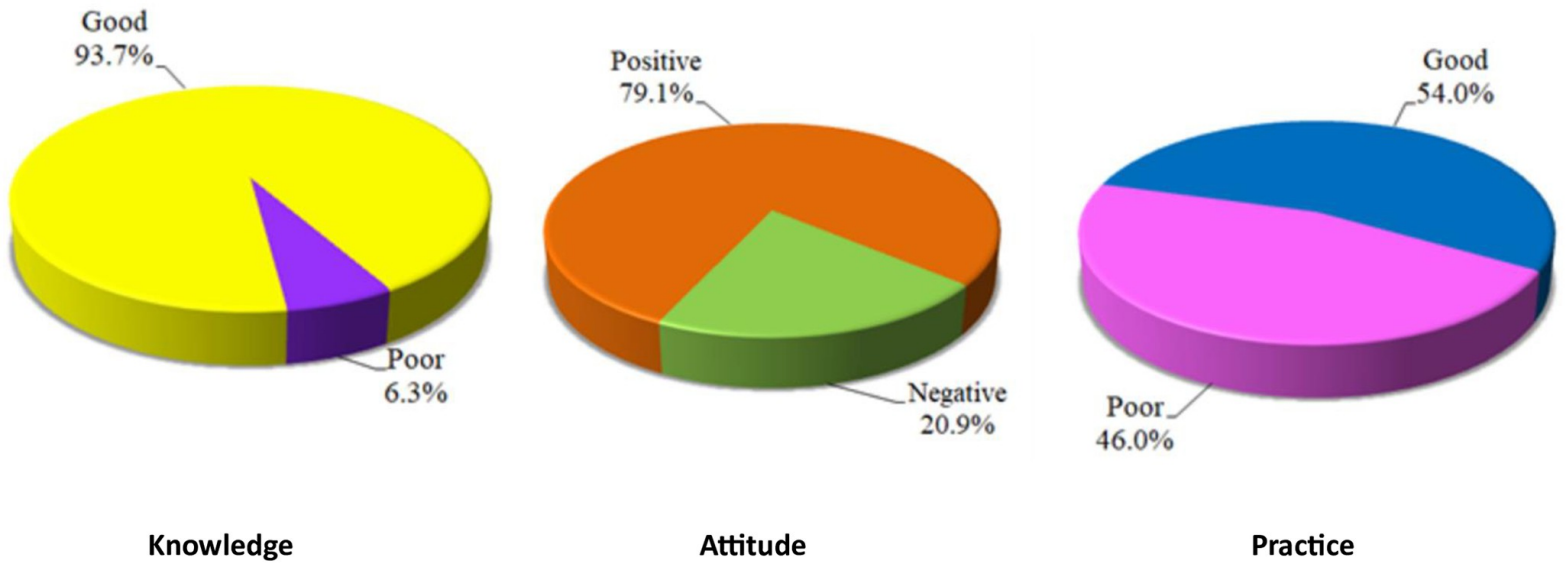
Distribution of level of knowledge, attitude and practice about antimicrobial prescription and resistance among the studied participants (n = 350).

The main sources of information about antibiotic prescription and resistance were international antibiotic guidelines, followed by advice from older colleagues and internet representing 21.1%, 17.7% and 13.7% respectively (Table 2). Most of HCPs (93.7%) recorded good knowledge level about antibiotic prescription and antimicrobial resistance with mean score of knowledge13.21 ± 1.83. About 79% of HCPs recorded a positive attitude towards proper antibiotic prescription with mean score of attitudes 63.02 ± 7.68. Fifty four percent of HCPs demonstrated a good level of practice with mean score of practice 9.75 ± 2.17. (Table 3; Fig 1).

**Table 2:** Distribution of the main sources of antibiotic information among the studied participants (n = 350).

The main source for antibiotic information	No.	%
Antibiotics policy in your local setting	36	10.3
Pharmaceutical companies	47	13.4
Conferences/seminars	17	4.9
National antibiotic Guidelines	45	12.9
Internet	48	13.7
International antibiotic guidelines	74	21.1
Advice from older colleagues	62	17.7
Advice from peers	18	5.1
Television	3	0.9

**Table 3:** Distribution of the mean scores of studied participants according to the level of knowledge, attitude and practice about antimicrobial prescription and resistance scores (n = 350).

	Total Score	% Score
**Knowledge**		
Mean ± SD.	13.21 ± 1.83	73.40 ± 10.16
Median (Min.–Max.)	13.0 (6.0–17.0)	72.22(33.33–94.44)
**Altitude**		
Mean ± SD.	63.02 ± 7.68	67.68 ± 11.29
Median (Min.–Max.)	64.0 (40.0–78.0)	69.12(33.82–89.71)
**Practice**		
Mean ± SD.	9.75 ± 2.17	60.93 ± 13.55
Median (Min.–Max.)	10.0 (4.0–15.0)	62.50 (25.0–93.75)

Regarding the relation between sociodemographic characteristics and level of knowledge, there were no statistically significant risk factors for bad knowledge level (Table 4). On the other hand, a statistically significant association was observed between positive attitude level and work institutions (X2 = 13.446, p = 0.021) (Table 5). Meanwhile, the relation between sociodemographic characteristics and level of practice, there were no statistically significant risk factors for poor practice level among HCPs (Table 6).

**Table 4:** Relation between sociodemographic characteristics and level of knowledge (n = 350).

Demographic characteristics	Knowledge				χ^2^	p
	Poor (n = 22)		Good (n = 328)			
	No.	%	No.	%		
**Sex**						
Male	15	68.2	161	49.1	3.008	0.083
Female	7	31.8	167	50.9		
**Age**						
<30	10	45.5	78	23.8	4.559	^MC^p = 0.171
30-<40	9	40.9	180	54.9		
40-<50	3	13.6	54	16.5		
≥50	0	0.0	16	4.9		
**Work Institution**						
Primary Health Care	0	0.0	17	5.2	5.086	^MC^p = 0.330
Ministry of Health and Population Hospital	7	31.8	58	17.7		
Health Insurance Organization	4	18.2	55	16.8		
University Hospital	8	36.4	163	49.7		
Private Hospital	3	13.6	23	7.0		
Private clinic	0	0.0	12	3.7		
**Educational Level**						
Bachelor	8	36.4	62	18.9	5.637	^MC^p = 0.199
Diploma	1	4.5	19	5.8		
Master	5	22.7	114	34.8		
Doctorate	3	13.6	83	25.3		
Fellowship	5	22.7	50	15.2		
**Work Experience**						
Less than 1 year	7	31.8	44	13.4	6.195	^MC^p = 0.089
1–5 years	4	18.2	51	15.5		
5 years—10 years	5	22.7	79	24.1		
More than 10 years	6	27.3	154	47.0		
**Did you attend any training/ educational course for antibiotics prescription**						
Yes	4	18.2	98	29.9	1.366	0.243
No	18	81.8	230	70.1		

χ^2^: Chi square test

MC: Monte Carlo

**Table 5:** Relation between sociodemographic characteristics and level of attitude among the studied participants (n = 350).

Demographic characteristics	Attitude				χ^2^	p
	Negative (n = 73)		Positive (n = 277)			
	No.	%	No.	%		
**Sex**						
Male	43	58.9	133	48.0	2.741	0.098
Female	30	41.1	144	52.0		
**Age**						
<30	25	34.2	63	22.7	4.109	0.250
30-<40	34	46.6	155	56.0		
40-<50	11	15.1	46	16.6		
≥50	3	4.1	13	4.7		
**Work Institution**						
Primary Health Care	5	6.8	12	4.3	13.446*	0.020*
Ministry of Health and Population Hospital	13	17.8	52	18.8		
Health Insurance Organization	3	4.1	56	20.2		
University Hospital	40	54.8	131	47.3		
Private Hospital	9	12.3	17	6.1		
Private clinic	3	4.1	9	3.2		
**Educational Level**						
Bachelor	21	28.8	49	17.7	5.384	0.250
Diploma	3	4.1	17	6.1		
Master	24	32.9	95	34.3		
Doctorate	17	23.3	69	24.9		
Fellowship	8	11.0	47	17.0		
**Work Experience**						
Less than 1 year	18	24.7	33	11.9	7.788	0.051
1–5 years	11	15.1	44	15.9		
5 years—10 years	14	19.2	70	25.3		
More than 10 years	30	41.1	130	46.9		
**Did you attend any training/ educational course for antibiotics prescription**						
Yes	23	31.5	79	28.5	0.250	0.617
No	50	68.5	198	71.5		

χ^2^: Chi square test MC: Monte Carlo

*: Statistically significant at p ≤ 0.05

**Table 6:** Relation between demographic characteristics and level of practice among the studied participants (n = 350).

Demographic characteristics	Practice				χ^2^	p
	Poor (n = 161)		Good (n = 189)			
	No.	%	No.	%		
**Sex**						
Male	84	52.2	92	48.7	0.425	0.514
Female	77	47.8	97	51.3		
**Age**						
<30	42	26.1	46	24.3	2.414	0.491
30-<40	91	56.5	98	51.9		
40-<50	21	13.0	36	19.0		
≥50	7	4.3	9	4.8		
**Work Institution**						
Primary Health Care	9	5.6	8	4.2	1.741	0.884
Ministry of Health and Population Hospital	30	18.6	35	18.5		
Health Insurance Organization	24	14.9	35	18.5		
University Hospital	79	49.1	92	48.7		
Private Hospital	14	8.7	12	6.3		
Private clinic	5	3.1	7	3.7		
**Educational Level**						
Bachelor	35	21.7	35	18.5	4.979	0.289
Diploma	7	4.3	13	6.9		
Master	62	38.5	57	30.2		
Doctorate	35	21.7	51	27.0		
Fellowship	22	13.7	33	17.5		
**Work Experience**						
Less than 1 year	25	15.5	26	13.8	2.036	0.565
1–5 years	27	16.8	28	14.8		
5 years—10 years	42	26.1	42	22.2		
More than 10 years	67	41.6	93	49.2		
**Did you attend any training/ educational course for antibiotics prescription**						
Yes	43	26.7	59	31.2	0.856	0.355
No	118	73.3	130	68.8		

χ^2^: **Chi square test**

## Discussion

The increase of antibiotic resistance is of a great global health concern. With the complexity of COVID19 pandemic, antibiotic resistance has increased due to the over and misuse of antibiotics. Although the diagnosis of COVID19 has been more efficient nowadays but antibiotic treatment is still being prescribed by HCPs as a precaution [31]. Health care practitioners are the cornerstone of good health systems. A well-educated and skilled workforce is the fundamental requirement to ensure healthy community, and this is more important during a crisis such as COVID-19 pandemic [32]. Thus, this study was designed to evaluate knowledge, attitude and practices for antimicrobial prescription and its resistance among health care providers in the COVID-19 era.

In poor and middle-income countries, insufficient training and education of HCPs regarding proper antimicrobial use is one of the most important risk factors that affects antimicrobial resistance [33]. In our study three quarters of HCPs (248) (70.9%) stated that they didn’t attend any training or educational course for antibiotics prescription. This result showed accordance with other study held in Egypt by El-Sokkary et al. as it revealed that (66.4%) of the participants did not get any training or educational courses about antibiotic prescription, although most of them have completed their postgraduate studies [28]. Also, a study by Olaru et al. in Zimbabwe stated that only third of participants (31%) reported having received training in antibiotic prescribing [34]. Higher results were reported in a study in Lebanon by Mina and Azakir, as 48.9% of healthcare practitioners had participated in antibiotic prescription trainings before the COVID-19 pandemic, and 39.3% participated in similar activity during the pandemic [35]. These results reflect low rates for attending courses and education programmes about antibiotic uses and resistance especially in Africa, so raising the awareness about implementing these courses should be encouraged in these countries.

The main sources of information about antibiotic prescription and resistance in our study were international antibiotic guidelines, followed by advice from older colleagues and internet representing 21.1%, 17.7% and 13.7% respectively. In a study held in Zimbabwe, they stated that national guidelines were the main source for guiding prescribing in routine practice (93%). Other sources of information to support prescribing were textbooks in (70%), discussions with colleagues (63%) and professional meetings (62%) [34].

Knowledge obviously reflects the practice of individuals as it is the base for good practice. The knowledge of HCPs regarding antibiotic use and resistance especially during COVID-19 era is of a great importance. Physicians, Pharmacists, and Dentists are the professionals who are in the front line having the authority to prescribe and give antibiotics to the patients, so they must be fully aware of the associated risks with this practice. In the present study most of the HCPs (93.7%) recorded good knowledge level about antibiotic prescription and antimicrobial resistance with mean score of knowledge13.21 ± 1.83. In a study carried out by Seaton et al. in United Kingdom, respondents reported strong knowledge on antimicrobial resistance and high trust in fulfilling their antibiotic stewardship programme within everyday practices (92.7%) [36]. Also, in a study that was conducted in Nepal by Cheoun et al. showed a high level of theoretical knowledge (87.8%) while low level of practical knowledge (22.5%) was found [37]. Medical doctors demonstrated the best knowledge of AMR (80% answered all questions correctly) in a study conducted by Ashiru-Oredope et al. [38]. Although most of our respondents stated that they didn’t attend any training or educational course for antibiotics prescription, good knowledge was recorded. This finding may be due to that more than half of our respondents (58.6%) had obtained Medical Decorate or Masters’ degree, most of them work in university hospitals and obtain good knowledge from their seniors and more than 69% of them have more than 5 years job experience.

Regarding the attitude dimension, in our study 79% of HCPs recorded a positive attitude towards proper antibiotic prescription with mean score of attitudes 63.02 ± 7.68. On the other side, lower levels of positive attitude were recorded (49%) by Nwafia et al. [39]. In Cheoun et al. study, the attitudes toward the severity of AMR and acceptability of AMR control were at a moderate level [37]. Statistically significant association was observed between positive attitude level and work institutions. This may be as a result of prescribing antibiotics by physicians according to their availability in the hospitals, the attitude of their seniors and the lack of antimicrobial stewardship in work institutes. Other study held in KSA identified the associated factors with positive attitude were age, nationality and qualifications [40]. However, a study that was conducted during COVID-19 did not reveal any association between positive attitudes and demographic characteristics [41].

Below half of our study participants of HCPs demonstrated a good level of practice. Other study by Muradyan et al. reported 63% good level of practice among practitioners [42]. Although good knowledge level was recorded in our study, practice level was noticed to be lower than it, wherein participants could correctly identify appropriate uses of antibiotics, and yet fail to apply these findings in practice. This finding may be due to the doctor’s perception of patient demand rather than actual patient needs. Patients’ fear during the COVID 19 pandemic led to their unwillingness to wait without taking antibiotics once they caught an infection. Physicians may prescribe antibiotics for patients in cases where most of the patients did not have secondary bacterial infection or a positive bacterial culture. Behavioural characteristics of physicians and patients lead to unnecessary antibiotic prescribing. Studies showed that physicians feel pressured to prescribe antibiotics due to patient preferences [43]. Also, workload on doctors may lead them not to wait for results of cultures and rush in prescribing antibiotics. A systematic review held by Sudhinaraset et al. finds that in developing countries providers recommend antibiotics due to convenience, affordability, and social and cultural preferences; many providers across countries reported poor adherence to national guidelines of antimicrobials using [44].

In order to improve a doctor’s attitude and practice towards incorrect prescribing, organised interventions are needed. In this situation, antimicrobial stewardship programmes (ASPs) can play a significant role in limiting the misuse of antibiotics in hospitals. However, it is unknown to what extent hospitals have implemented standardized ASPs, and there is a lack of key implementation data and facts on the rollout of ASPs, particularly in low- and middle-income countries. Hospitals lack the tracking and documenting of antibiotic usage for the purpose of submitting it to a provincial, national, or international database [45]. The CDC stated two perspectives to evaluate the antimicrobial stewardship, i.e., institutional policies and commitment (facility indicator) and individual attitude and practises (physician indicators) [46]. In our study, although HCPs reported good knowledge of antibiotic use, lacking good practices may be due to the absence of successful ASP application. A study that was held in Egypt reported that more than half of surveyed hospitals did not have an official antibiogram [47]. Another study held by EL-Sokkary et al. found that (22.6%) only reported they have implemented ASP at their workplace and (13.4%) had antibiotic lists classified into “Access, Watch, Reserve” groups [28]. These low rates highlight the requirement for current national data on the difficulties and obstacles associated with adopting ASP in Egyptian hospitals. It is possible to conduct a thorough assessment of the precise causes and allocate resources to expand the implementation coverage which will eventually lead to proper practices of antibiotics using.

## Strength and limitation of the study

The study included different specialties of health care providers from several work institutions. The study used a validated data collection tool. The limitation that data was collected online due to precautions of COVID19 using a convenient sampling method which might affect generalization of our results. The relatively small sample size, which may not accurately represent the actual KAP of physicians in EGYPT as a whole. Although, Participants were from 12 governorates, a larger sample size and random sample would have been preferable to produce a more broadly applicable result. The need for Internet access to complete the questionnaire was yet another restriction because not all HCPSs had enough time during the COVID 19 pandemic to access the internet to fill in the questionnaire which might resulted in selection bias. A cross-sectional survey cannot clarify the cause-effect relationship. The study didn’t assess some variables that may affect prescription practice such as workload, shifts and work stress during the Covid19 pandemic. Almost all participants were physicians. The sample didn’t include different categories of HCPs.

## Conclusion and recommendations

Health care practitioners in our study have a good level of knowledge and attitude about antibiotics however lower level of proper practice towards the problem of AMR in COVID19 era was noticed. Low rates for attending courses and education programmes about antibiotic uses and resistance were found. Implementation of effective policies and guidelines is crucial to evaluate the antimicrobial use especially in the COVID-19 era to reduce the unintended consequences of the misuse of antibiotics and its impact on AMR. Moreover, healthcare institutes must ensure that healthcare practitioners receive training and education for proper antimicrobial prescribing. The bulk of the essential components of antimicrobial stewardship are either missing or "Under Process," which points to serious gaps in the practice. If ignored, these gaps will cause severe problems and ultimately have an impact on how effectively antibiotics are used in hospitals. Stakeholders should implement antibiotic stewardship programmes quickly in hospitals to stop the spread of antibiotic resistance and improve antibiotic therapy. Evaluation of the barriers is needed to make future plans on improving awareness of and access to antibiotic resources, encouraging HCWs to use these resources and encouraging them not to prescribe when they feel antibiotics are not required. Behavioural change strategies should be implemented to reach effective and positive behaviours towards antibiotics use.
